# Hospitals *V*. School Clinics

**Published:** 1915-09-25

**Authors:** 


### THE MEDICAL INSPECTION OF SCHOOL CHILDREN.

### Hospitals v. School Clinics.

NOTWITHSTANDING the disorganisation consequent upon the war, the medical inspection and medical treatment of school children under the supervision of the Board of Education goes on increasing in range and in utility, as may be easily apprehended from a study of the annual report of the Board's chief medical officer (Sir George Newman, M.D.), published this year at a considerably earlier date than is usual. The report deals with many branches of the work that is being done, and is everywhere informative and instructive; especially is this the case with the sections dealing with the organisation and results of treatment, in which so many of the voluntary hospitals of the kingdom are taking a considerable share. In the opinion of the chief medical officer, however, the hospitals do not offer advantages for his purpose so great as can often be obtained by other agencies. Thus he states definitely that the "school clinic" (of which 179 were sanctioned for the year under review, ending March 31, 1915) still maintains its position as the most generally suitable institution for dealing with children suffering from remediable defects. Still, Sir George concedes that, "making every allowance for the limitations necessary in hospital treatment, it must be said that these institutions have played, and must continue to play, a great part in providing treatment that would otherwise be totally unavailable."

### WHAT THE HOSPITALS ARE DOING.

Let us see what it is the hospitals are actually doing for the Board, or rather for the elementary school children for whom the Board is responsible. Contributions to hospitals were sanctioned by the Board in seventy-five areas, fifteen of which were counties. A long table showing the arrangements made and the nature of the work done is set forth on pp. 100, 101, 102 of the report; but it does not include, of course, the large number of hospitals which are daily treating school children quite apart from any formal scheme of a local education authority: as part of their ordinary charitable work, that is, without grants in aid from the public funds. It is important that this distinction should be borne in mind, for the hospitals are actually doing a vast work for these children, of which no details are presented to the Board's cognisance or are recognised in any official report.

As regards the system of subsidising grants to the hospitals which are officially recognised and utilised by the Board, considerable variety is apparent both in the methods of payment adopted and in the adequacy of such payment. Sir George Newman definitely states that "in a large proportion of the areas where the local education authority contributes to the hospital, this contribution can in no sense be considered as an adequate remuneration for work done; it is, rather, in the nature of a subscription in grateful recognition of the large amount of good work voluntarily undertaken by hospitals on behalf of school children." This frank admission of a plain fact which has frequently been insisted on in these columns in previous years must be gratifying to the voluntary hospital managers; but it is a confession that some portion of the work of the Board of Education is being paid for out of private charity—that is to say, out of the revenues of the voluntary hospitals and out of the underpaid or unpaid labours of their medical staffs.

### A PROPER BUSINESS BASIS.

On the other hand, says the report, there are certain areas in which the hospital authorities require a fee per case which is at least equivalent to the cost to the hospital of such treatment. In other words, there are certain hospitals which have insisted on a proper business basis for their co-operation with the Board and do not make a loss on the transaction. This seems to the outside observer to be the more excellent way; and if some hospitals have got backbone enough to compel justice from the Board we do not know why the rest should not follow the same example. Between the two extremes—that of recompense adequate enough to avoid actual loss to the hospital and that of a "contribution which can in no sense be considered as adequate remuneration for work done"—various arrangements, both as to method and amount, for payments to hospitals are found in practice.

### THE SCHOOL CLINIC.

Upon the old question of the relative efficiency of the hospital and the school clinic, Sir George Newman has little to add this year to his previous pronouncements. He is an adherent of the clinic as against the hospital, and he summarises the old argument briefly as follows. The possession of suitable premises and of the necessary, possibly costly, equipment may constitute the hospital the most suitable place for treatment of certain conditions, especially when the numbers to be dealt with are small. On the other hand, it must be remembered, especially in regard to conditions requiring continuous treatment or subsequent supervision, that hospital treatment is seldom complete; and children who have undergone treatment at a hospital will frequently require the further treatment or supervision which can only be given adequately at the school clinic. Thus in Derbyshire, where the system of clinics has been extended, "it has been found in the past that when treated at hospitals scattered in various parts of the county they have attended in the out-patient departments, and the officers in these out-patient departments are too busy to follow the cases up in a satisfactory manner." It is especially in regard to minor ailments that this criticism of the hospitals is uttered—such, for instance, as adenoids, scabies, impetigo, conjunctivitis, ringworm, and so on. With regard to ringworm, the Board's sheet anchor is still the $x$-ray treatment. The figures show a progressive decrease in the number of cases under treatment each year; and though the rate of diminution is not rapid, it is at least satisfactory as far as it goes. There appears to be a prejudice on the part of parents against this treatment, due perhaps to the occasional unlucky cases in which alopecia is produced.

London a Special Case.

In London a comprehensive treatment scheme in connection with the hospitals was adopted three years ago by the London County Council, and a special addendum to Sir George Newman's report deals with the working of the scheme. It will be recalled that the agreements with the twelve hospitals and the thirty-four treatment centres provide for the payment to the hospital or centre of £50 a year for each doctor, surgeon, or anæsthetist working on one half-day a week, and *pro rata* for each half-day worked. In addition a capitation payment is made of two shillings for each major ailment treated in respect of the eye, ear, nose and throat, and teeth, and for the *x*-ray treatment of ringworm a capitation fee of seven shillings is paid. Details are given of the various hospitals and centres, and of the number of children which each is prepared to undertake. The treatment is in the hands of 157 doctors and 37 dentists, but some of the former hold dual appointments at more than one centre. Sir George Newman does not express any opinion as to the comparative value of this London scheme, and of those in operation elsewhere; but the voluminous extracts given from the School Medical Officer's report (to the London County Council) convey the impression that the work is being thoroughly well done.

